# Comparison of 27-Gauge and 25-Gauge Microincision Vitrectomy Surgery for the Treatment of Vitreoretinal Disease: A Systematic Review and Meta-Analysis

**DOI:** 10.1155/2020/6149692

**Published:** 2020-08-18

**Authors:** Jinlan Ma, Qing Wang, Haoyu Niu

**Affiliations:** ^1^Department of Ophthalmology, Affiliated Hospital of Qinghai University, No. 29 of Tongren Road, Xining 810000, Qinghai Province, China; ^2^Medical College of Qingha University, No. 16 of Konglun Road, Xining 810000, Qinghai Province, China

## Abstract

**Background:**

We performed a systematic review and meta-analysis to evaluate the safety and effectiveness of 27-gauge (27-G) microincision vitrectomy surgery (MIVS) compared with 25-guage (25-G) MIVS for the treatment of vitreoretinal disease.

**Methods:**

A systematic electronic search was conducted in March 2020 in PubMed, Embase, and the Cochrane library. Eligible criteria for including studies were controlled trials comparing 27-G vitrectomy with 25-G vitrectomy in patients with vitreoretinal disease. The main outcomes included operation time; best corrected visual acuity (BCVA) in logMAR; postoperative intraocular pressure (IOP); primary anatomical success rate for rhegmatogenous retinal detachment (RRD) cases and postoperative central macular thickness (CMT) for idiopathic epiretinal membrane (ERM) cases; intraoperative/postoperative complications. Odds ratio (OR) and mean difference (MD) were synthesized under fixed or random effects models.

**Results:**

Eleven studies enrolling 940 eyes were identified. Among those 11 studies, six studies were on the treatment of RRD and five studies were on the treatment of ERM, so subgroup analyses were conducted. The total pooled results indicated that 27-G surgery system had obvious advantages in improving BCVA at six months after the vitrectomy (*P* = 0.004) and reducing intraoperative/postoperative complications (*P* = 0.03). However, the mean operation time was significantly longer by three minutes for 27-G compared with 25-G vitrectomy (*P* = 0.002). In subgroup analyses, for the treatment of ERM cases, 27-G group was associated with less complications and longer operation time. However, for the treatment of RRD cases, 27-G groups and 25-G groups were comparable in operation time, postoperative BCVA, postoperative IOP, and primary anatomical success rate.

**Conclusions:**

This meta-analysis confirmed that 27-G MIVS was an effective and safe surgical system compared with 25-G MIVS for the treatment of RRD and ERM cases, even though 27-G system needs a longer surgical time.

## 1. Introduction

Microincision vitrectomy surgery (MIVS) was first introduced by Machemer in the early 1970s [1], and this technique represented a new era in ophthalmology. Since that moment, 20-gauge (20-G), 23-G, and 25-G surgery system were applied to vitrectomy [2–4]. There was no doubt that current MIVS with 25-G or 23-G instruments had simplified vitrectomy procedure and had provided numerous potential advantages over traditional 20-G surgery [5–10]. It was confirmed that smaller wounds could reduce the intraocular inflammation [9, 11], shorten recover time [7, 12], and decrease intraoperative/postoperative complications [12, 13]. In 2010s, Oshima et al. first described 27-G MIVS for the treatment of vitreoretinal diseases [14]. In the beginning, the 27-G vitrectomy was conducted mainly for simple cases such as epiretinal membrane (ERM), idiopathic macular holes, and vitreous hemorrhage [15–17]; recently, indications for 27-G vitrectomy have since been expanded to more complicated conditions, including proliferative diabetic retinopathy, rhegmatogenous retinal detachment (RRD), and proliferative vitreoretinopathy [18–20]. Many studies have shown the advantages of 27-G MIVS in terms of patients' comfort, convalescence, inflammatory response, and visual recovery in the ERM surgery compared with traditional 25-G vitrectomy [15, 17, 21–23]. However, some studies concluded that 27-G vitrectomy requires longer operation time for the treatment of RRD cases because of the lower flow rate. Moreover, 27-G vitrectomy induced more postoperative inflammation because of sutureless wounds [20, 24–26].

We performed this meta-analysis to evaluate the feasibility, safety, and effectiveness of 27-G instrument for uncomplicated macular diseases such as ERM and complicated peripheral vitreoretinal disorders such as RRD. This study would expand our current knowledge of the safety and effectiveness of the 27-G MIVS.

## 2. Methods

### 2.1. Search Strategy and Inclusion Criteria

This meta-analysis was conducted in accordance with the Cochrane Handbook for Systematic Reviews of Interventions and Preferred Items for Systematic Reviews and Meta-Analysis (PRISMA) Statement. Two researchers independently performed the literature search in the PubMed, the Cochrane Library, and EMBASE database until March 2020. The search used the following keyword strings: “27-gauge,” “25-gauge,” and “vitrectomy” in various combinations with the language limited to English. The reference lists of case reports, studies, and review articles were also reviewed for any additional citations. To increase sample size, we included both control trials and observational studies. Studies that appeared twice, or focused on other outcomes based on the same study group, were removed as the duplicated publications. All relevant articles identified through the search were scanned based on the title, keywords, and abstract by at least two investigators and were rejected in the initial screening if the article clearly did not meet the inclusion criteria. When a title/abstract could not be rejected with certainty, full texts of retrieved publications were reviewed and evaluated.

### 2.2. Inclusion Criteria

Studies were included if they (i) compared 27-G with 25-G vitrectomy for vitreoretinal disease, (ii) randomized controlled trials (RCTs), cohort, case-control or cross-sectional studies with at least four weeks' followup, and (iii) contained sufficient information of treatment outcome.

### 2.3. Data Extraction

The following information were extracted by the investigators independently from the published reports, using a standardized protocol and reporting form: first author's last name, year of publication, country of origin, number of enrolled eyes, mean age of patients, vitreoretinal disease, followup information, and related complications.

### 2.4. Outcome Measures

The main outcomes for this meta-analysis included operation time; best corrected visual acuity (BCVA) in logMAR at six months post-vitrectomy (POM6); intraocular pressure (IOP) at postoperative day 1 (POD1); primary anatomical success rate for RRD cases; postoperative central macular thickness (CMT) for ERM cases and intraoperative/postoperative complications. Complications were defined as adverse events result from surgery such as hypotony, intraocular hypertension, wound suture because of leakage, recurrent RD, vitreous hemorrhage (VH), iatrogenic retinal breaks (IRBs), and other surgery-related complications.

### 2.5. Assessment of Methodology Quality

Reviewers independently assessed the qualities of the included trials using a system which was previously reported by Downs and Blacks [27]. This system was appropriate for both randomized and nonrandomized studies. The system comprises 27 items distributed among five subscales regarding reporting (10 items), external validity (3 items), bias (7 items), confounding (6 items), and power (1 item). The total maximum score was 31. The studies with a quality score of more than 16 were considered to have adequate quality. Any discrepancy in the quality assessment between the two observers was discussed and a consensus was reached.

### 2.6. Statistical Analysis

Data were processed by REVMAN (Version 5.3; The Cochrane Collaboration, Copenhagen, Denmark). We calculated the mean difference (MD) for the continuous outcome along with 95% confidence intervals (CIs) by inverse variance method. For discontinuous outcomes, the summary odds ratios (ORs) were calculated by Mantel–Haenszel method. *P* < 0.05 was considered statistically significant, and 95% confidence intervals (CIs) were reported.

The between-study heterogeneity was tested by the chi-square-based (*χ*^2^) Cochran's statistics and the inconsistency index (*I*^2^) [28], which indicated the proportion of variability across studies due to heterogeneity rather than sample error. In the presence of substantial heterogeneity (*I*^2^ > 45%), the random effect model (REM) was adopted as the pooling method; otherwise, when *I*^2^ < 45%, the fixed effect model (FEM) was adopted as the pooling method.

The leave-one-out sensitivity analysis was performed using *I*^2^ > 50% as the criteria for evaluating the key studies with a substantial impact on between-study heterogeneity. Subgroup analyses were conducted for RRD cases and ERM cases in order to reduce heterogeneity. A funnel plot was performed to look for evidence of publication bias. The funnel plot should be asymmetric when there is publication bias or symmetric in the case of no publication bias.

## 3. Results

### 3.1. Literature Search

**Figure 1:**
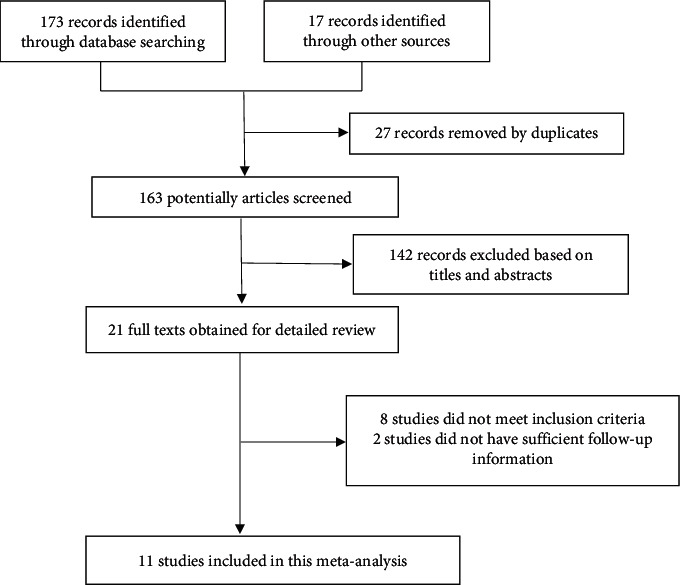
Flow diagram of literature search and study selection.

A total of 190 studies were initially identified. The abstracts were reviewed, and 21 studies with potentially relevant trials were reviewed in their entirety. Subsequently, eight studies were excluded because they did not have sufficient followup information and two studies were excluded because of design heterogeneity (Figure 1). Finally, a total of 11 studies were included in this meta-analysis.

### 3.2. Characteristics and Quality of Included Studies

Table 1 lists the characteristics of the included studies. These studies were published between 2015 and 2019. Seven studies [15, 21–24, 26, 31] were designed as prospective randomized/nonrandomized comparative study and four studies [17, 25, 29, 30] were retrospective randomized/nonrandomized comparative study. Five trials [15, 17, 21, 24, 29] enrolled 554 eyes which were conducted on ERM and six studies [22, 23, 25, 26, 30, 31] enrolled 386 eyes which were conducted on RRD. In total, 940 eyes were included in this meta-analysis; 439 eyes were assigned to the 27-G vitrectomy group and 501 eyes to the 25-G group. The duration of followup ranged from one to twelve months. The mean age in each study was not significant difference between the 27-G and 25-G group. All the patients underwent vitrectomy with Constellation vision system.

For the Downs and Blacks score, all studies were over 16 which means the quality of these studies were adequate.

### 3.3. Operation Time

**Figure 2:**
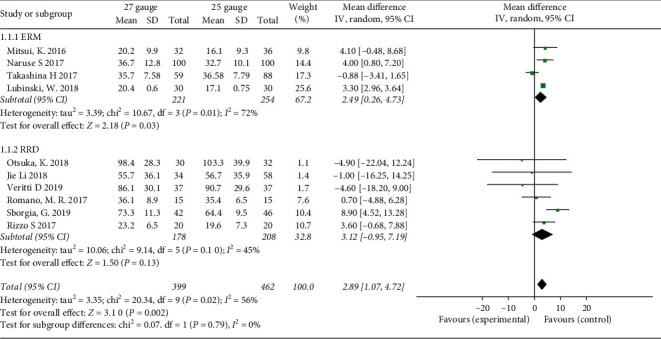
Forest plots of operation time compared between 27-G and 25-G vitrectomy in overall and subgroup analysis. RRD: rhegmatogenous retinal detachment; ERM: epiretinal membrane.

Ten studies compared operation time between 27-G and 25-G group, the total pooled result showed that 27-G vitrectomy needs approximately three minutes longer than 25-G, and the difference was statistically significant (MD = 2.89; 95% CI: 1.07, 4.72; *P* = 0.002) (Figure 2). There was moderate heterogeneity among studies (*I*^2^ = 56%, *P* = 0.02), and so, REM meta-analysis was used. In order to explore the potential sources of heterogeneity, sensitivity analysis (via excluding the studies one by one) was proceeded. After the Takashina et al. study [17] was excluded, the heterogeneity almost disappeared (*I*^2^ = 19%, *P* = 0.27; MD = 3.64; 95% CI: 2.29, 4.99; *P* < 0.001), which indicated this study can be identified as the main contributor of heterogeneity. We reevaluated the study of Takashina et al. in terms of design, statistics, and selection bias and did not find anything wrong. In fact, this study did not influence the final pooled result.

Subgroup analyses were conducted on RRD and ERM. For the treatment of ERM, the operation time was approximately 2.5 min longer in 27-G group, and the difference was significant (MD = 2.49; 95% CI: 0.26, 4.73; *P* = 0.01). However, the difference was not significant for the treatment of RRD (MD = 3.12; 95% CI: −0.95, 7.19; *P* = 0.13).

### 3.4. Visual Outcome

**Figure 3:**
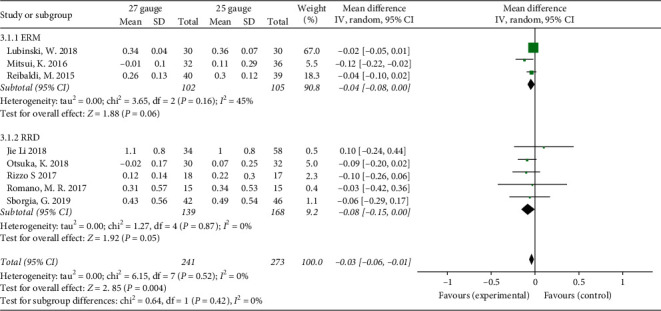
Forest plots of best corrected visual acuity (BCVA) compared between 27-G and 25-G vitrectomy at 6 months postoperative in overall and subgroup analysis.

Data on BCVA were provided in eight studies. The pooled result indicated that the 27-G group had a favorable response in visual recovery at six months after vitrectomy, and the difference was significant (MD = −0.03; 95% CI: −0.06, −0.01; *P* = 0.004) (Figure 3). Random effect model was adopted as the pooling method because of obvious heterogeneity in the ERM subgroup (*I*^2^ = 45%). In ERM subgroup, BCVA at six months after vitrectomy was comparable between 27-G group and 25-G group (MD = −0.04; 95% CI: −0.08, 0.00; *P* = 0.06). The sensitivity analysis showed the study of Mitsui K was the main source of heterogeneity. However, there was no statistics and selection bias in this study. In the RRD subgroup, there was no obvious difference between 27-G group and 25-G group (MD = −0.08; 95% CI: −0.15, 0.00; *P* ≥ 0.05, *I*^2^ = 0%).

### 3.5. IOP

**Figure 4:**
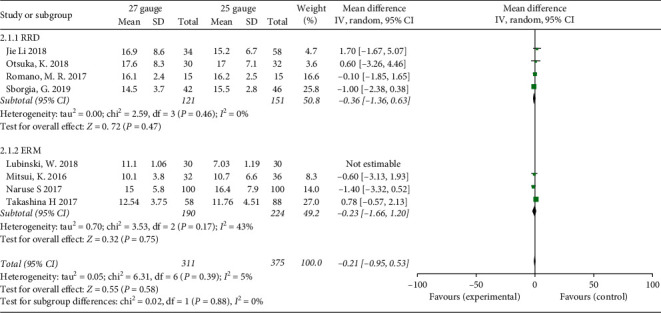
Forest plots of intraocular pressure (IOP) at POD1 compared between 27-G and 25-G vitrectomy in overall and subgroup analysis. POD1: postoperative day one.

Eight studies recorded IOP on the first day postoperative (POD1). 27-G group and 25-G group had the same effect in controlling postoperative IOP and there were no significant differences (MD = 0.53; 95% CI: −1.49, 2.54; *P* = 0.61). In the subgroup analysis, IOP on POD1 were comparable between 27-G and 25-G group for the RRD cases (MD = −0.36; 95% CI: −1.36, 0.63; *P* = 0.47) and ERM cases (MD = 0.83; 95% CI: −2.05, 3.7; *P* = 0.57). Significant heterogeneity was found (*I*^2^ = 92%), so random effects were used. The study of Lubinski et al. [24] was the main contributor of heterogeneity, and after reevaluating this study, we found nothing wrong (Figure 4).

### 3.6. Primary Anatomical Success Rate

**Figure 5:**
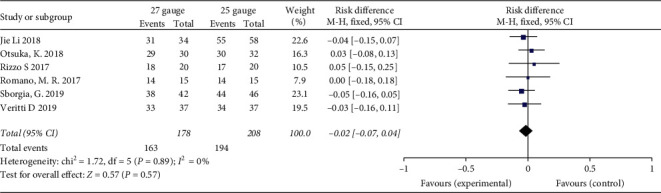
Forest plot of primary anatomical success rate compared between 27-G and 25-G vitrectomy for rhegmatogenous retinal detachment (RRD) cases.

The primary anatomical success rate after a single operation was 91.6% and 93.3% in the 25-G and 27-G groups, respectively (Figure 5). The pooled result indicated that there was no significant difference in primary anatomical success rate between 27-G and 25-G group for the treatment of RRD without obvious heterogeneity (OR = 0.8, 95% CI: 0.38, 1.71; *P* = 0.57; *I*^2^ = 0%).

### 3.7. CMT

**Figure 6:**
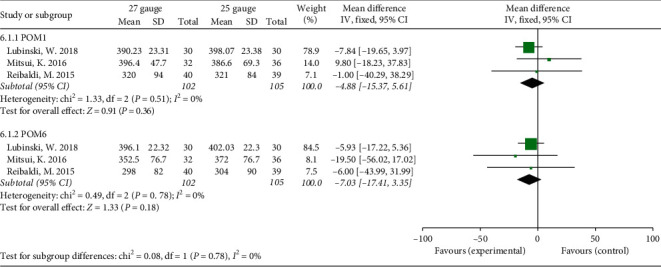
Forest plots of central macular thickness (CMT) at one month and six months postoperatively compared between 27-G and 25-G vitrectomy for epiretinal membrane (ERM) cases.

Three studies provided information on CMT with at least six months' follow-up. This meta-analysis collected data on CMT at one month (POM1) and six months postoperatively (POM6). The pooled result showed there were no significant differences in CMT for the treatment of ERM between the 25-G and 27-G groups during followup (1 month: *P* = 0.36; 6 months: *P* = 0.18, resp.) (Figure 6). No heterogeneity was found (*I*^2^ = 0%).

### 3.8. Intraoperative and Postoperative Complications

**Figure 7:**
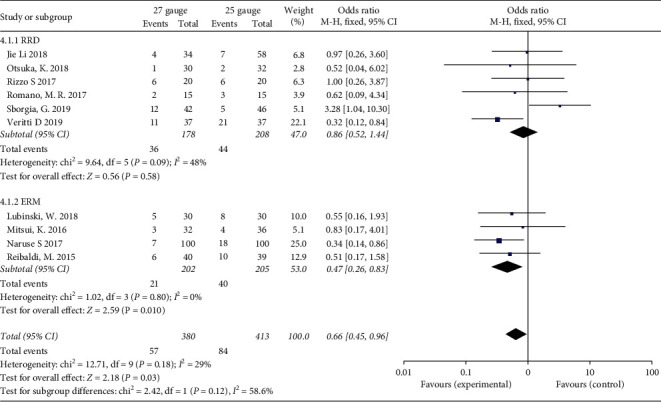
Forest plots of intraoperative and postoperative complication between 27-G and 25-G vitrectomy in overall and subgroup analysis. RRD: rhegmatogenous retinal detachment; ERM: epiretinal membrane.

Complications in each involved study were summarized in Table 1. Ten studies (6 on RRD and 4 on ERM) reported intraoperative and postoperative complications during followup. In the total analysis, 27-G group was associated with less complication compared with the 25-G group with a pooled OR of 0.66 (95% CI: 0.45 to 0.93, *P* = 0.03). However, the difference was not significant in the RRD cases in the subgroup analysis with a pooled OR of 0.86 (95% CI: 0.52, 1.44; *P* = 0.58). No significant heterogeneity was found (*I*^2^ = 29%) (Figure 7).

## 4. Discussion

To the best of our knowledge, this is the first meta-analysis to assess the effectiveness and safety of 27-G MIVS compared with 25-G MIVS for vitreoretinal disease, although only data on the treatment of ERM and RRD were available.

The pooled result illustrated that the time for performing 27-G vitrectomy was longer than that for 25-G vitrectomy. The difference between the two groups was attributed to the different internal diameters of the vitrectomy probe of the two surgery systems used. Some studies concluded that when comparing 27-G, 25-G, 23-G, and 20-G vitrectomy, more time is required for vitreous excision as the instrument gauge decreases [9, 12]. However, other studies reported the difference in operation time primarily due to the substantially lower infusion and aspiration rate of the 27-G vitrectomy system used in the present studies but not distinct instrument gauges [14, 15]. The difference of operation time was significant in the ERM subgroup, but not in the RRD subgroup, which can be explained by three reasons. First, in the RRD surgery, peripheral vitrectomy was more strenuous and complicated than in ERM surgery, and as a result, the operation time mostly depends on the proficiency of surgeon rather than the instrument gauges. Second, Veritti et al. [26] reported that the 27-G probe has excellent fluidics procedures and high cut rate (7500 cpm) and it is very effective in shaving peripheral vitreous for RRD cases, so the operation time was not prolonged by smaller gauge in 27-G group. Third, although the qualities of included studies were adequate and the sensitive analysis minimized the heterogeneity as much as possible, the heterogeneity in the ERM subgroup may influence the final result.

Regarding the relationship between gauge of instrument and postoperative BCVA, our study showed that BCVA was significantly better in the 27-G group at six months after vitrectomy compared with the 25-G groups. It is hard to explain the reason for this difference. With small gauge vitrectomy, significant inflammation and astigmatism is rarely seen at six months after the operation. For the treatment of RRD, early visual recovery is limited by the using of gas or silicone oil. However, most of the silicone oil was removed before six months after surgery. Also, there were no difference in CMT during followup, so postoperative macular edema should not play a role in visual recovery.

Postoperative IOP at POD1 in 27-G group were as stable as 25-G group. Postoperative hypotony induced by leakage of sutureless wound still remains a major complication that can lead to underfilling of tamponade, choroidal detachment, and endophthalmitis. However, Takashina et al. [17] reported that hypotony is usually transient and, in most cases, resolved spontaneously due to small gauge in 27-G vitrectomy. Furthermore, it was suggested that surgeons use oblique incisions and displacement of the conjunctiva to reduce wound leakage and stabilize postoperative IOP.

Operation effectiveness is one of the major theoretical concerns regarding 27-G instrument. Romano et al. [23] reported that the lower flow rate of 27-G system may influence the operation effectiveness. However, Veritti et al. [26] reported that dual pneumatically operated vitrectomy probes of 27G system with ultrahigh cut rates (7500 cpm) can maintain an efficient vitreous flow rate. For RRD cases, the primary anatomical success rate of included studies ranged from 89% to 97% using 27-G and 85% to 96% using 25-G MIVS and did not differ significantly between two groups. For ERM cases, there were no differences in postoperative CMT between 27-G and 25-G groups during the six-month follow-up period. This relationship suggests that 1 mm diameter reduction of sclerotomy in 27-G MIVS, as compared with 25-G MIVS, has no influence on the recovery of normal retinal structure in the vitreoretinal surgery and the 27-G was as effective as the 25-G system.

Speaking of the safety of the 27-G vitrectomy system, besides being less invasive when compared with 25-G system, 27-G carries additional potential advantages; the shorter but flexible vitrectomy probe generates the shortest attraction distance and a smaller “sphere of influence.” This allows a more accurate fluid control and a greater dissection precision, theoretically allowing for safer procedures with less intro- and postoperative complications compared with 25-G system [26].

The first point of strength in this meta-analysis was that the measurement of outcomes was fairly consistent and pooled results should not be biased due to misclassification. The second point of strength was that the likelihood of bias was minimized by performing a meticulous search for published studies and using explicit methods for study selection, data extraction, quality assessment, and statistical analysis. Third, subgroup and sensitivity analyses were used to confirm the reliability of the pooled results.

This meta-analysis has several potential limitations that should be taken into account. First, the main limitation of this review is the small number of RCTs. Second, we cannot fully exclude publication bias. Third, our analysis was based on only 11 trials, and most of them have a small sample size, which can affect the interpretation of the results. Fourth, some results were limited by heterogeneity between the involved trials.

## 5. Conclusions

In conclusion, our data demonstrated that although 27-G vitrectomy need longer operation time, it had obvious advantages in reducing complications compared with 25-G system for the treatment of ERM. However, these features were not obvious for the treatment of RRD cases. Multicenter controlled trials should be conducted to determine the overall long-time benefits of 27-G vitrectomy for the treatment of all kinds of vitreoretinal disease.

## Figures and Tables

**Table 1:** Characteristics of involved studies.

Included studies	Design	Disease	Location	No. of eyes 27G/25G	Age (year) mean ± SD 27G/25G	Followup (months)	Complications (eyes) 27G/25G
Reibaldi et al. [21]	Prospective randomized comparative study	ERM	Italy	40/39	66 ± 6/64 ± 6 *P* = 0.14	12	IRB: 0/3 VH: 2/3 Hypotony: 1/2 ERM recurrence: 3/2
Mitsuiet al. [15]	Prospective nonrandomized comparative study	ERM	Japan	32/36	68.9 ± 5.3/65.4 ± 11.4 *P* = 0.33	6	Hypotony: 3/4
Takashina et al. [17]	Retrospective randomized comparative study	ERM	Japan	59/88	72.9 ± 6.3/71.3 ± 7.9 *P* : NA	1	NA
Rizzo et al. [9]	Prospective nonrandomized comparative study	RRD	Italy	20/20	64.7 ± 9.7/62.4 ± 9.8 *P* : NA	6	IRB: 2/1 Choroidal detachment: 1/0 RD: 2/3 Intraocular hypertension: 1/2
Naruse et al. [29]	Retrospective nonrandomized clinical trial	ERM	Japan	100/100	67.6 ± 9.6/69.4 ± 8.9 *P* = 0.25	6	RD: 0/1 VH: 1/1 Hypotony: 2/6 Intraocular hypertension: 4/10
Romanoet al. [23]	Prospective randomized comparative study	RRD	Italy	15/15	58 ± 8/59 ± 11 *P* = 0.82	6	RD: 1/1 VH: 1/0 Intraocular hypertension: 0/2
Liet al. [30]	Retrospective nonrandomized clinical trial.	RRD	China	34/58	58.5 ± 13.3/54.1 ± 12.5 *P* = 0.1	6	RD: 3/3 IRB: 1/2 Hypotony: 0/1 Intraocular hypertession: 0/1
Lubinski et al [24]	Prospective randomized comparative study	ERM	Poland	30/30	65.40 ± 4.29/67.50 ± 4.18 *P* = 0.052	6	RD: 1/1 Macular hole: 1/0 Hypotony: 3/7
Otsuka et al. [25]	Retrospective nonrandomized clinical trial	RRD	Japan	30/32	59 ± 13/55 ± 9 *P* = 0.15	6	RD: 1/2
Sborgia et al. [31]	Prospective randomized comparative study	RRD	Italy	42/46	59.9 ± 9.2/61.7 ± 8.7 *P* = 0.35	12	RD: 4/2 Choroidal detachment: 1/0 ERM: 5/2 CME: 2/1
Veritti et al. [26]	Prospective randomized comparative study	RRD	Italy	37/37	63.9 ± 13.5/63.1 ± 12.5 *P* = 0.8	6	RD: 4/3 Hypotony: 0/2 Intraocular hypertension: 4/5 Wound suture: 3/11

CME: cystoid macular edema; ERM: epiretinal membrane; IRBs: iatrogenic retinal breaks; RRD: rhegmatogenous retinal detachment; VH: vitreous hemorrhage; hypotony was defined as IOP < 6 mmHg and intraocular hypertension was defined as IOP > 21 mmHg.

## Data Availability

The data used to support the findings of this study are available from the corresponding author upon request.
